# Repurposing immunosuppressants for antileukemia therapy

**DOI:** 10.15252/emmm.202217042

**Published:** 2022-12-01

**Authors:** Maiko Sezaki, Gang Huang

**Affiliations:** ^1^ Department of Cell Systems and Anatomy UT Health San Antonio Joe R. & Teresa Lozano Long School of Medicine San Antonio TX USA; ^2^ Department of Pathology and Laboratory Medicine UT Health San Antonio Joe R. & Teresa Lozano Long School of Medicine San Antonio TX USA; ^3^ Mays Cancer Center at UT Health San Antonio San Antonio TX USA

**Keywords:** Cancer, Haematology

## Abstract

Drug repurposing, the strategy to identify new therapeutic use for clinically approved drugs has attracted much attention in recent years. This strategy offers various advantages over traditional approaches to develop new drugs, including shorter development timelines, low cost, and reduced risk of failure. In this issue of *EMBO Molecular Medicine*, Liu *et al* show that inosine monophosphate dehydrogenase (IMPDH) inhibitors, the well‐known immunosuppressants have a potent therapeutic effect on the aggressive blood cancer, acute myeloid leukemia with MLL rearrangements. Intriguingly, the antileukemia effect of IMPDH inhibitors is mediated, at least in part through the overactivation of TLR signaling and Vcam1 upregulation. The robust antileukemia effect of IMPDH inhibitors, both *in vitro* and *in vivo*, together with their mechanistic findings provides a rational basis for repurposing IMPDH inhibitors for antileukemia therapy.

## Graphical abstract

G Huang and M Sezaki discuss repurposing of immunosuppressants targeting IMPDH for treatment of acute myeloid leukemia as reported by S Goyama *et al*, in this issue of *EMBO Mol Med*.

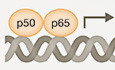

Acute myeloid leukemia (AML) is characterized by unrestricted proliferation of myeloid progenitor cells. Patients with AML undergo a standard treatment regimen, which was first described in the 1970s, typically involving high doses of cytarabine combined with anthracyclines. However, the treatment landscape for AML has changed substantially in the past 5 years with the emergence of new molecularly targeted drugs, including venetoclax to target BCL2, midostaurin and gilteritinib to target FLT3, and ivosidenib and enasidenib to target mutant IDH1/2 (DiNardo & Wei, 2020). Repurposed drugs present a promising source of agents with potential to further improve the outcome of AML patients (Wojcicki *et al*, 2020). The repurposing candidates for leukemia therapy should have well‐defined targets, favorable pharmacokinetic profiles, and differential toxicity between leukemia cells and normal hematopoietic cells.

Accumulation of chromosome aberrations and/or gene mutations in hematopoietic stem and progenitor cells lead to AML development. The *MLL* gene (also known as *KMT2A*) located on chromosome 11q23 is involved in chromosomal translocations in a subtype of AMLs (Yokoyama, 2015). Over 130 different MLL translocations with numerous different fusion partners, including AF9, ENL, and AF4 have been described. MLL‐rearranged proteins promote the self‐renewal capabilities of hematopoietic stem and progenitor cells through constitutive activation of *MLL* target genes, such as *HOXA9* and *MEIS1*. MLL‐rearranged AML is associated with aggressive progression and monocytic or myelomonocytic differentiation, which corresponds to FAB AML‐M4 or AML‐M5 morphologies. Despite the increasing number of new drugs and treatment regimens, patients with MLL‐rearranged leukemia generally have poor prognosis in comparison to those with other AML subtypes. Therefore, there is a clear unmet need for novel therapeutic approaches to treat MLL‐rearranged AML.

Inosine monophosphate dehydrogenase (IMPDH) is a rate‐limiting enzyme in *de novo* guanine nucleotide biosynthesis (GMP, GDP, and GTP), which catalyzes the conversion of inosine monophosphate (IMP) to xanthosine monophosphate (XMP; Naffouje *et al*, 2019). Guanine nucleotides play a vital role in diverse signaling pathways and biological processes. They are biosynthesized through salvage and *de novo* synthesis pathways in cells. The salvage pathway reproduces guanine nucleotides using the already assembled nucleobases (guanine, hypoxanthine) or nucleoside (guanosine). The key enzyme in the salvage pathway is HRPT1, which catalyzes the reaction of guanine or hypoxanthine with PRPP to form GMP or IMP, respectively. The salvage GTP biosynthesis pathway is particularly important in the brain, but is insufficient to satisfy the needs of most dividing cells. Therefore, rapidly proliferating cells such as lymphocytes and cancer cells largely depend on the *de novo* guanine nucleotide synthesis pathway to sustain their growth. Two isoforms of IMPDH, IMPDH1 and IMPDH2 have been identified. They share 84% identical amino acid sequences and are indistinguishable in their enzymatic activity. IMPDH1 is generally expressed at lower levels except in certain tissues, while IMPDH2 is the predominant isoform in most proliferating cells. The elevated activity of IMPDH2, observed in a variety of cancer cells makes this isoform a promising therapeutic target.

IMPDH inhibitors have been used clinically for decades as safe and effective immunosuppressants (Naffouje *et al*, 2019; Valvezan *et al*, 2020). The first IMPDH inhibitor, mycophenolic acid (MPA) was identified in fungi more than 100 years ago. Subsequent studies revealed its function in inhibiting IMPDH as well as its immunosuppressive properties. MPA's success in multiple clinical trials, together with its orally bioavailable prodrug, mycophenolate mofetil (MMF) resulted in their FDA approval as immunosuppressants during solid organ transplantation. Mizoribine is another IMPDH inhibitor used predominantly in Asia for autoimmune disorders and for preventing organ rejection after transplantation. Ribavirin is also an FDA‐approved purine analog, which is mainly used as an antiviral agent. FF‐10501‐01 is a prodrug of mizoribine and was shown to have anticancer effects in several preclinical studies. In addition to their immunosuppressive function, the antitumor activity of IMPDH inhibitors has been known since the late 1960s. Based on promising results from preclinical studies, several clinical trials were conducted to test their efficacy against myeloid tumors and pancreatic ductal carcinoma. Unfortunately, none of them were successful, primarily due to the short duration of response and their toxicity, and consequently, there are no FDA‐approved IMPDH inhibitors for cancer therapy. Nevertheless, several recent studies that use modern molecular technologies to show their antitumoral properties have sparked renewed interest in IMPDH inhibitors for anticancer therapy (Kofuji *et al*, 2019).

In this issue of *EMBO Molecular Medicine*, Liu *et al* (2022) report the remarkable therapeutic potential of two IMPDH inhibitors, MPA and FF‐10501‐01 for MLL‐rearranged AML. They first showed that human AML cell lines and patient‐derived primary AML cells with MLL rearrangements are particularly susceptible to IMPDH inhibitors. They then assessed the *in vivo* effects of IMPDH inhibitors using an AML mouse model driven by MLL‐AF9, and confirmed their robust ability to suppress the development of MLL‐AF9 leukemia *in vivo*. Of note, Liu *et al* administered the IMPDH inhibitors every 2 days to the leukemia mice. The “alternate day” administration of FF‐10501‐01 had no devastating effects on immune cells and efficiently inhibited MLL‐AF9‐induced leukemogenesis. The “alternate day” administration, instead of the standard daily administration protocol might be worth testing in future clinical trials.

As a mechanism of action, Liu *et al* proposed that IMPDH inhibition induces overactivation of TLR signaling in AML cells. Using reporter cell lines that express individual TLRs and the NF‐κB‐GFP reporter, they found that MPA significantly increased TLR‐mediated NF‐κB activation. MPA treatment also increased TNF‐α and IL‐1β expression in bone marrow‐derived macrophages, and enhanced IκBα degradation and p38 phosphorylation in MLL‐AF9 cells. TLR overactivation resulted in the upregulation of Vcam1, a key cell adhesion molecule involved in inflammation, which promoted excessive cell–cell interaction of AML cells and prevented their efficient proliferation. Furthermore, co‐treatment with FF‐10501‐01 and the TLR1/2 agonist showed a stronger antileukemia effect than FF‐10501‐01 alone. These results suggest that IMPDH inhibitors suppress leukemogenesis partly through the overactivation of TLR signaling and Vcam1 upregulation. However, the detailed mechanism of IMPDH‐mediated regulation of TLR signaling remains unknown. Given that the MPA‐induced activation of TLR signaling was reversed by guanosine supplementation, it appears that TLR signaling is activated in response to a decrease in guanine nucleotides. The potential crosstalk between GTP levels and TLR‐mediated innate immune responses could be an interesting topic for future research. It is also unclear why IMPDH inhibitors are particularly effective in MLL‐rearranged AML, despite the high expression of TLR1, TLR2, and TLR4 in some non‐MLL‐rearranged AMLs. Of note, Liu *et al* also found that IMPDH inhibition induced the activation of the p53‐p21 pathway and downregulation of the CD98/Lat1‐mTORC1 pathway, both of which have been shown to be effective in suppressing the development of MLL‐rearranged AML (Bajaj *et al*, 2016; Hayashi *et al*, 2019). Although p53 inhibition alone appears insufficient to cancel the effects of IMPDH inhibitors as previously reported (Kofuji *et al*, 2019), these changes could also contribute to the antileukemia properties of IMPDH inhibitors (Fig. 1).

**Figure 1:**
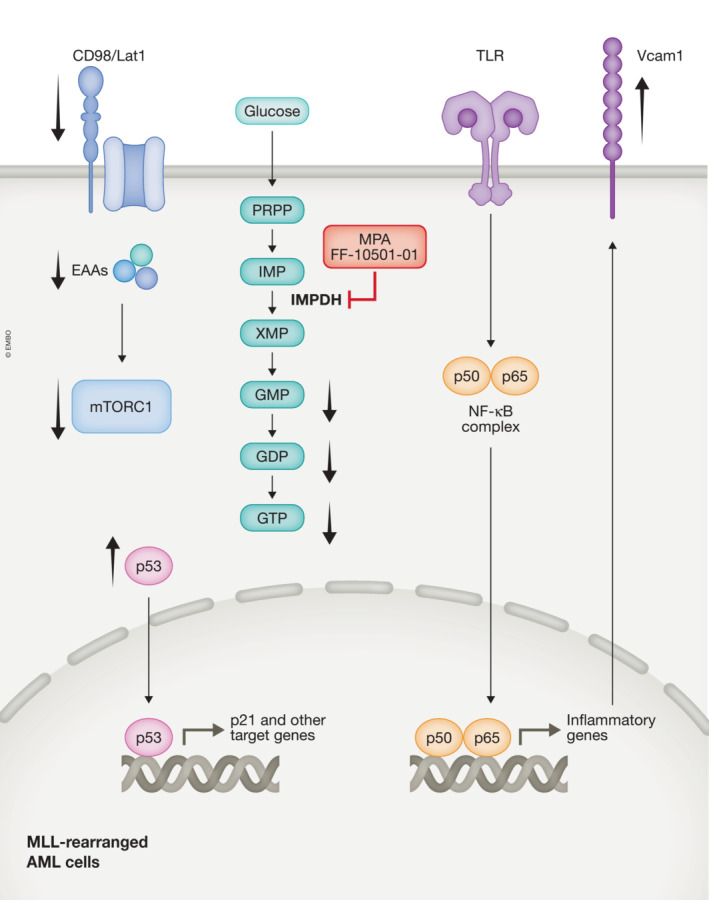
The diverse effects of IMPDH inhibitors in MLL‐rearranged AML IMPDH is a rate‐limiting enzyme in the *de novo* GTP synthesis pathway that catalyzes the conversion of IMP to XMP. MPA and FF‐10501‐01 are IMPDH inhibitors used in this study. In addition to the reduction of guanine nucleotides (GMP, GDP, and GTP), IMPDH inhibition induces diverse metabolic and epigenetic changes in MLL‐rearranged AML cells, such as downregulation of CD98/Lat1 and mTORC1 signaling, activation of p53‐p21 pathway, activation of TLR‐NF‐κβ signaling, and Vcam1 upregulation. EAAs, essential amino acids; IMPDH, inosine monophosphate dehydrogenase.

Several unanswered mechanistic questions remain, but the results presented in this study highlight the potential in repurposing IMPDH inhibitors toward antileukemia therapy. Given the critical role of IMPDH in T and B lymphocytes, immunologically “cold tumors” including leukemia and glioblastoma would be most responsive to IMPDH inhibitors *in vivo*. Future studies should identify biomarkers to predict the response of each tumor to IMPDH inhibition. It is also important to develop combination therapies with IMPDH inhibitors. In addition to the TLR1/2 agonist, which was shown in this study to be effective in treating MLL‐AF9 leukemia, IMPDH inhibitors could be combined with other molecularly targeted drugs. Interestingly, Liu *et al* found that MPA synergized with the BCL2 inhibitor, venetoclax to inhibit the growth of AML cells *in vitro*. Although venetoclax‐based combination therapy has significantly improved outcomes for AML patients, it has become apparent that monocytic AMLs are relatively resistant to venetoclax (Pei *et al*, 2020). As monocytic AMLs often express high levels of TLRs, targeting IMPDH could overcome this resistance. The work by Liu *et al* will guide future investigations to repurpose IMPDH inhibitors to treat patients with AML.
